# Chronic Low-Level Lead Exposure Increases Mesenteric Vascular Reactivity: Role of Cyclooxygenase-2-Derived Prostanoids

**DOI:** 10.3389/fphys.2020.590308

**Published:** 2021-01-07

**Authors:** Maylla Ronacher Simões, Bruna Fernandes Azevedo, María Jesús Alonso, Mercedes Salaices, Dalton Valentim Vassallo

**Affiliations:** ^1^Department of Physiological Sciences, Federal University of Espirito Santo, Vitória, Brazil; ^2^Health Science Center of Vitória-EMESCAM, Vitória, Brazil; ^3^Department of Basic Health Sciences, Rey Juan Carlos University, Alcorcón, Spain; ^4^Department of Pharmacology, School of Medicine, Autonomous University of Madrid, Hospital La Paz Institute for Health Research (IdiPaz), Madrid, Spain

**Keywords:** lead exposure, cyclooxygenase-2, vascular reactivity, mesenteric arteries 2, peripheral vascular resistance

## Abstract

Lead (Pb) exposure causes hazardous effects as hypertension and other cardiovascular diseases. We evaluated whether chronic Pb exposure alters the peripheral vascular resistance measuring the vascular reactivity of mesenteric resistance arteries in rats to identify the underlying mechanisms that are associated to the development of Pb-induced hypertension. Mesenteric resistance arteries from lead-treated and untreated Wistar rats (1st dose: 10 μg/100 g; subsequent doses: 0.125 μg/100 g, intramuscular, 30 days) were used. Contractile responses to phenylephrine increased, while acetylcholine and sodium nitroprusside-induced relaxation was not affected by lead treatment. Endothelium removal and inhibition of NO synthase by L-NAME similarly enhanced the response to phenylephrine in untreated and lead-treated rats. The antioxidants apocynin and superoxide dismutase (SOD) did not affect vasoconstriction in either group. The vascular expression of cyclooxygenase-2 (COX-2) protein increased after lead exposure. The respective non-specific or specific COX-2 inhibitors indomethacin and NS398 reduced more strongly the response to phenylephrine in treated rats. Antagonists of EP1 (SC19220), TP (SQ29548), IP (CAY10441) and angiotensin II type 1 (losartan) receptors reduced vasoconstriction only in treated rats. These conclusions present further evidence that lead, even in small concentration, produces cardiovascular hazards being an environmental contaminant that account for lead-induced hypertension.

## Introduction

Lead is an environmental and industrial pollutant without a biological role. It exerts toxic effects on several organs and systems of the organism, including the development of hypertension (Xie et al., 1998). Several reports suggest that it contributes to the genesis and/or maintenance of hypertension increasing hemodynamic parameters and peripheral vascular resistance. Functional changes such as increased sympathetic activity and renin-angiotensin system and insulin resistance are also involved in humans (Freis, 1973; Harrap, 1994). More recently, the participation of the immune system and inflammatory mechanisms has also been demonstrated in mice (Trott and Harrison, 2014). But only recently the role of toxic metals has aroused the curiosity of the scientific world in the genesis of hypertension (for reviews see Prozialeck et al., 2008; Vassallo et al., 2011; Shakir et al., 2017).

Several mechanisms have been implicated in lead-induced hypertension, which might increase vascular peripheral resistance. Among them are the inhibition of Na, K-ATPase (Weiler et al., 1990; Fiorim et al., 2012), the reduction of nitric oxide (NO) bioavailability and the increased endothelial release of endothelin (Khalil-Manesh et al., 1993; Gonick et al., 1997; Grizzo and Cordelline, 2008; Vaziri and Gonick, 2008; Silveira et al., 2014); the participation of free radicals by reducing NO bioavailability (Vaziri et al., 2001; Vaziri, 2002); depletion of antioxidant reserves (Farmand et al., 2005; Patrick, 2006) and increase of ROS production (Farmand et al., 2005). In addition, studies in rats have shown the increase plasma angiotensin-conversing enzyme activity might be implicated in the endothelial dysfunction associated with the lead-induced hypertension (Carmignani et al., 1999; Simões et al., 2011; Silveira et al., 2014). Another mechanism involved in the lead-induced hypertension in rats is the increase of sympathetic nerve activity followed by the reduction of baroreflex sensitivity and parasympathetic tone (Carmignani et al., 1999; Simões et al., 2016).

We also emphasize that lead exposure at low blood level concentration increase the reactivity of the aorta by reducing NO bioavailability and increasing ROS and COX-2-derived prostanoids (Silveira et al., 2014; Simões et al., 2015). In addition, it is already known that COX-2-derived prostanoids contribute to the altered vascular responses in hypertensive animals (Alvarez et al., 2005; Wong et al., 2010; Martínez-Revelles et al., 2013) and also show that COX-2 is a source of reactive oxygen species (ROS) in vessels (Martínez-Revelles et al., 2013; Virdis et al., 2013). Reinforcing such mechanism several studies demonstrated that angiotensin II modulates prostaglandin production by regulating COX-2 expression in rat aorta vascular cells (Ohnaka et al., 2000; Alvarez et al., 2007; Beltrán et al., 2009). The renin-angiotensin system also plays a role since losartan treatment reduced the production of COX-2-derived products (Alvarez et al., 2007).

Clinical and experimental studies provide evidence that exposure to Pb is a risk factor in the development of hypertension (Vaziri, 2002; Vupputuri et al., 2003; Rahman et al., 2006; Fiorim et al., 2011; Silveira et al., 2014; Simões et al., 2015). Recently we demonstrated that chronic exposure to lead increased blood pressure in rats with blood levels below the recommended limits (Simões et al., 2015). Thus, the underlying mechanism involved in the increase of reactivity in small arteries, the main cause of hypertension, also remains to be elucidated. This study investigates the role of oxidative stress, COX-2 and its derived prostanoids, and angiotensin II in the vascular reactivity changes in mesenteric resistance arteries induced by 30-day treatment with a low lead concentration.

## Materials and Methods

### Ethics Statement and Animals

Male Wistar (250–300 g) rats were obtained from the Animal Quarters of the Health Center of the Federal University of Espírito Santo (CCS-UFES). All experimental procedures were conducted according to the research guidelines established by the Brazilian Societies of Experimental Biology and were approved by the institutional Ethics Committee in Animal Research of the Federal University of Espírito Santo (CEUA 063/2011).

Rats were housed under a 12-h light/12-h dark cycle, with free access to water and were fed with rat chow *ad libitum*. Rats were randomly distributed in two groups: control (vehicle-saline, intramuscular) or treated with lead acetate for 30 days (1st dose: 10 μg/100 g; subsequent doses: 0.125 μg/100 g, intramuscular, to cover daily loss) according to the model of Simões et al. (2015). The doses were adjusted weekly based on the weights of the rats and all animals survived at the end of the treatment. At the end of the treatment, the rats were killed by exsanguination after being anesthetized with intraperitoneal doses of ketamine (50 mg/kg) and xylazine (10 mg/kg). Thereafter, the mesenteric arteries were carefully dissected, the third-order mesenteric resistance arteries (MRA) were selected, the fat and connective tissue were removed. In sequence they were placed in Krebs-Henseleit solution (KHS, in mM: 115 NaCl, 25 NaHCO_3_, 4.7 KCl, 1.2 MgSO_4_ 7H_2_O, 2.5 CaCl_2_, 1.2 KH_2_PO_4_, 11.1 glucose, and 0.01 Na_2_EDTA) at 4°C.

### Vascular Function

For the vascular reactivity experiments, the MRA were divided into cylindrical segments of 2 mm in length and mounted in a wire myograph for the measurement of isometric tension (Model Myo Tech Danish, Model 410A and 610M, JP-Trading I/S, Aarhus, Denmark) (Mulvany and Halpern, 1977). The segments were stretched to their optimal lumen diameter for active tension development. This value has been set based on the internal circumference-to-wall tension ratio of each segment by setting their internal circumference (Lo) to 90% of what the vessels should have if exposed to a passive tension equivalent to that produced by a transmural pressure of 100 mm Hg. A 45 min equilibration period was taken before MRA were exposed to 120 mM KCl to assess their functional integrity. The presence of endothelium has been confirmed by the acetylcholine (Ach, 10 μM) induced relaxation attaining approximately 50% of the contraction induced by 120 mM KCl, in arteries pre-contracted with phenylephrine. Segments with endothelium were used to perform all experiments. Concentration–response curves to ACh (0.1 nM–100 μM) or sodium nitroprusside (0.1 nM–300 μM) were then performed in arteries previously contracted with phenylephrine at a concentration that produced 50% of the contraction to KCl in each case. After a 60 min washout, concentration–response curves to phenylephrine (0.1 nM–300 μM) were constructed. Single curves were performed on each segment. The effects of NG-nitro-L-arginine methyl ester (L-NAME, a non-specific NO synthase (NOS) inhibitor, 100 μM), apocynin (antioxidant, presumed NADPH oxidase inhibitor, and 30 μM), superoxide dismutase (SOD, 150 U/mL), indomethacin (non-specific COX inhibitor, 10 μM), NS398 (COX-2 inhibitor, 1 μM), SC19220 (EP1 receptor antagonist, 1 μM), SQ29548 (TP receptor antagonist, 1 μM), CAY10441 (IP receptor antagonist, 100 nM) and losartan (angiotensin II type 1 receptor antagonist, 10 μM) were investigated after their addition to the organ bath 30 min before performing the phenylephrine concentration-response curve. The endothelium dependency of the response to phenylephrine was investigated after its mechanical removal by rubbing the lumen with a horse hair. The inability of 10 μM Ach to produce relaxation confirmed the absence of endothelium.

### Western Blot Analysis

Frozen samples of MRAs were sonicated with ice-cold RIPA buffer (Sigma Aldrich, St Louis, MO, United States). The lysate was centrifuged at 6,000 rpm, the supernatant of soluble proteins was collected, and the protein concentration was determined by Lowry assay. Laemmli solution was added to aliquots containing 80 μg of protein from each animal. The proteins were separated on a 10% SDS-polyacrylamide gel and blotted to PVDF membrane (Amersham, GE Healthcare, Buckinghamshire, United Kingdom). Blots were incubated overnight at 4°C with mouse monoclonal antibodies for COX-2 (1:200; Cayman Chemical, Ann Arbor, MI, United States). Membranes were washed and incubated with a horseradish peroxidase-coupled anti-mouse (1:5,000; Stress Gen Bioreagent Corp., Victoria, BC, Canada) antibody for 1 h at room temperature. After thoroughly washing, the bands were detected using an ECL plus Western blotting detection system (GE Healthcare) after exposure to X-ray AX film (Hyperfilm ECL International). Blots were quantified using the Image J densitometry analysis software (National Institutes of Health). Anti α-actin (1:5,000, Sigma Chemical Co.) expression was used as a loading control.

### Drugs and Reagents

L-phenylephrine hydrochloride, acetylcholine chloride, sodium nitroprusside, L-NAME, apocynin, indomethacin, SOD, losartan, salts and other reagents were purchased from Sigma Chemical Co., and Merck (Darmstadt, Germany). NS398, SQ29548, SC19220, and CAY10441 were purchased from Cayman Chemical (Ann Arbor, MI, United States). Lead acetate was obtained from Vetec (Rio de Janeiro, RJ, Brazil). All drugs were dissolved in distilled water except NS398, SC19220, and CAY10441, which were dissolved in DMSO, and SQ29548, which was dissolved in ethanol. DMSO and ethanol did not have any effects on the parameters evaluated for vascular reactivity.

### Data Analysis and Statistics

The tension developed by the MRAs were expressed as a percentage of the maximal response induced by 120 mM KCl. Relaxation responses to ACh or SNP were expressed as the percentage of the previous contraction. The maximal effect (R_*max*_) and the concentration of agonist that produced 50% of the maximal response (EC_50_) were calculated for each concentration-response curve, using non-linear regression analysis (Graph Pad Prism 6, Graph Pad Software, Inc., San Diego, CA, United States). The sensitivities of the agonists were expressed as pD_2_ (−log EC_50_). The differences in the area under the concentration response curves (dAUC) for the control and experimental groups were used to compare the effects of endothelium denudation, L-NAME and indomethacin, on the contractile responses to phenylephrine. AUCs were calculated from the individual concentration-response curve plots using a computer program (GraphPad Prism 6, Graph Pad Software, Inc., San Diego, CA, United States). Differences were expressed as the percentage of the AUC of the corresponding control situation.

Data were expressed as the mean ± SEM of the number of animals in each experiment. The data was evaluated using Student’s *t*-test or one- or two-way ANOVA, followed by the Bonferroni *post hoc* test or *Tukey*’s test, using Graph Pad Prism Software. Differences were considered significant at *P* values equal to or <0.05.

## Results

Lead acetate exposure for 30 days attained blood lead levels of 21.7 ± 2.38 μg/dL, with similar body weight [Ct: before 218 ± 3.08 g and after 30 days 325 ± 5.80 g (*n* = 9); Pb: before 217 ± 2.57 g and after 30 days 328 ± 7.27 g (*n* = 9) *P* > 0.05] and presenting increased systolic blood pressure (Ct: 127 ± 0.57 mmHg, *n* = 7; Pb: 144 ± 1.67 mm Hg, *n* = 7, *P* < 0.05), as previously reported (Simões et al., 2015).

### Effects of Lead Treatment on Vascular Reactivity

**FIGURE 1:**
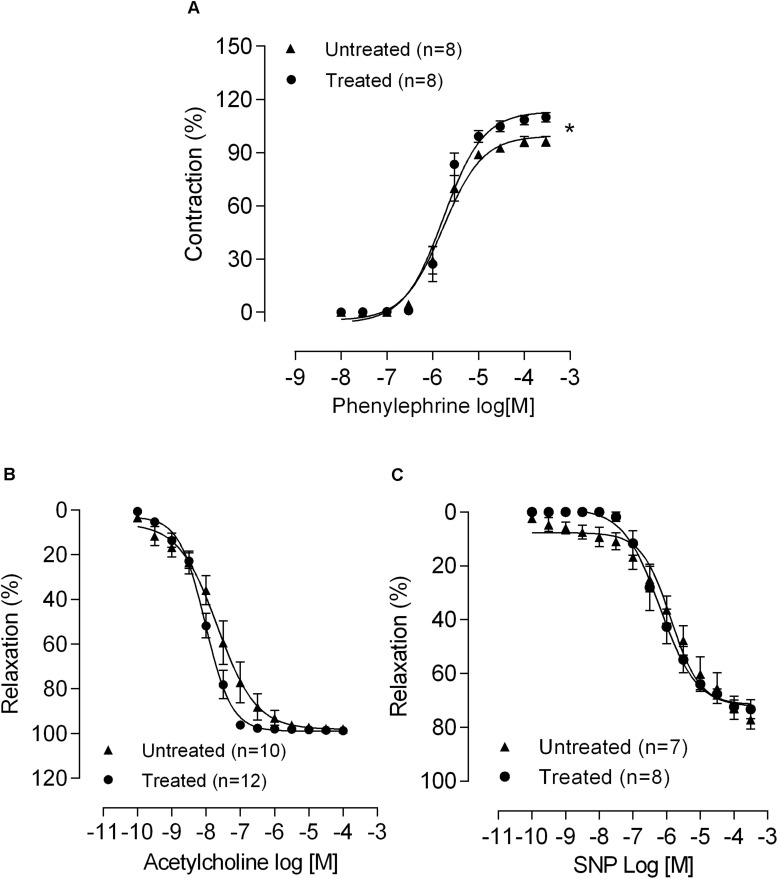
Chronic lead treatment affects MRA reactivity. The effects of 30 days of exposure to lead on the concentration-response curves to **(A)** phenylephrine, **(B)** acetylcholine, and **(C)** sodium nitroprusside (SNP). Data are expressed as the mean ± SEM. *P < 0.05 versus untreated using two-way ANOVA and Bonferroni *post hoc* test. n denotes the number of animals used.

Response to KCl was not affected by lead treatment in mesenteric arteries (untreated: 2.12 ± 0.09 mN/mm, *n* = 11; lead-treated: 2.39 ± 0.13 mN/mm, *n* = 11; *P* > 0.05). However, vasoconstrictor responses to phenylephrine increased (Figure 1A and Table 1). The Ach-induced vasodilator responses (Rmax, Ct: 97,78 ± 0.86 *n* = 10, Pb: 98.73 ± 0.61% *n* = 12; EC50, Ct: −7.78 ± 0.38 *n* = 10, Pb −8.07 ± 0.06, *n* = 12) and SNP (Rmax, Ct: 77.13 ± 3.57 *n* = 5, Pb: 73.31 ± 3.47% *n* = 4; EC50, Ct: −5.78 ± 0.3 *n* = 5, Pb: −6.19 ± 2.23 *n* = 4) were unaffected by lead treatment (Figures 1B,C), suggesting that the metal did not affect the endothelial function of the mesenteric rings.

**TABLE 1:** pD_2_ and the maximum response to phenylephrine in mesenteric segments from untreated rats and rats treated with lead with or without endothelium, L-NAME, apocynin, SOD, indomethacin, NS398, SC19220, SQ29548, CAY10441, or losartan.

	Untreated		Lead treated	
	R_*max*_	Pd_2_	R_*max*_	pD_2_
Control	96 ± 2.7	−5.72 ± 0,08	110 ± 2.6*	−5.79 ± 0.08
E-	114 ± 7.7*	−5.84 ± 0.24	108 ± 3.3	−6.28 ± 0.09†
L-NAME	111 ± 3.5*	−6.32 ± 0.15*	124 ± 4.6†	−6.33 ± 0.09†
Apocynin	103 ± 1.3	−5.32 ± 0.07	102 ± 4.5	−5.71 ± 0.08
SOD	101 ± 3.8	−5.42 ± 0.09	101 ± 3.0	−5.58 ± 0.07
Indomethacin	91 ± 1.7	−5.23 ± 0.08*	87 ± 5.4†	−5.26 ± 0.15†
NS398	94 ± 3.2	−5.61 ± 0.13	99 ± 2.4†	−5.52 ± 0.10
SC19220	101 ± 4.7	−5.47 ± 0.13	81 ± 3.4†	−5.01 ± 0.09†
SQ29548	94 ± 4.4	−5.73 ± 0.13	98 ± 2.9†	−5.6 ± 0.12
CAY10441	103 ± 4.5	−6.06 ± 0.17	99 ± 3.26†	−5.65 ± 0.30
Losartan	100 ± 4.7	−5.65 ± 0.10	93 ± 4.44†	−5.21 ± 0.14

Data are expressed as the mean ± SEM. R_max_ values are expressed as a percentage of the maximal response induced by 75 mM KCl. *P < 0.05 versus control untreated. †P < 0.05 versus control lead-treated. R_max_, Maximal response.

### Effects of Lead Treatment on the Endothelial Modulation of Vasoconstrictor Responses

**FIGURE 2:**
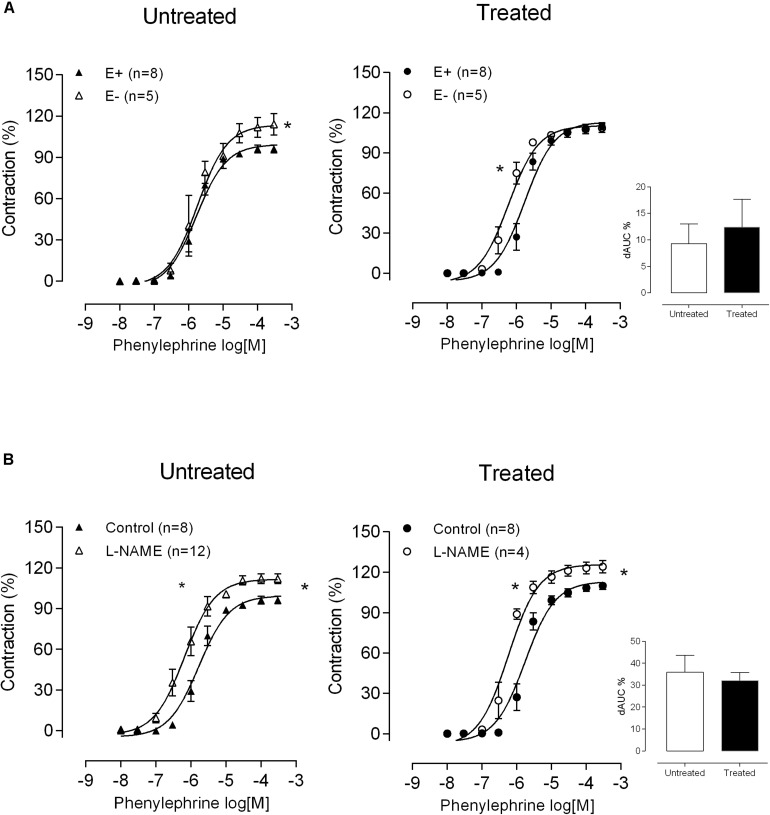
Role of nitric oxide in altered phenylephrine responses after lead treatment. The effects of **(A)** endothelium removal (E^–^) and **(B)** L-NAME (100 μM) on the concentration-response curve to phenylephrine in mesenteric rings from untreated and treated rats. *P < 0.05 versus E + or control using two-way ANOVA and Bonferroni post-test. The insert shows differences in the area under the concentration-response curves (dAUC) in denuded and intact endothelium segments and in the presence and absence of L-NAME. *P < 0.05 versus untreated by Student’s *t*-test. Data are expressed as the mean ± SEM. n denotes the number of animals used.

To investigate whether lead treatment could alter the NO modulation of MRA, the effects of endothelium removal and incubation with the NOS inhibitor L-NAME (100 μM) on vasoconstrictor responses to phenylephrine were investigated. Both protocols, the endothelium removal and L-NAME incubation caused a leftward shift in the concentration-response curves to phenylephrine in mesenteric segments from both groups. A similar effect was found in both the untreated and treated groups, as shown by the dAUC values (Figures 2A,B). These findings suggest that endothelial NO production and/or bioavailability remained unaffected after lead treatment.

### Role of Oxidative Stress in Lead Effects on Vasoconstrictor Responses

**FIGURE 3:**
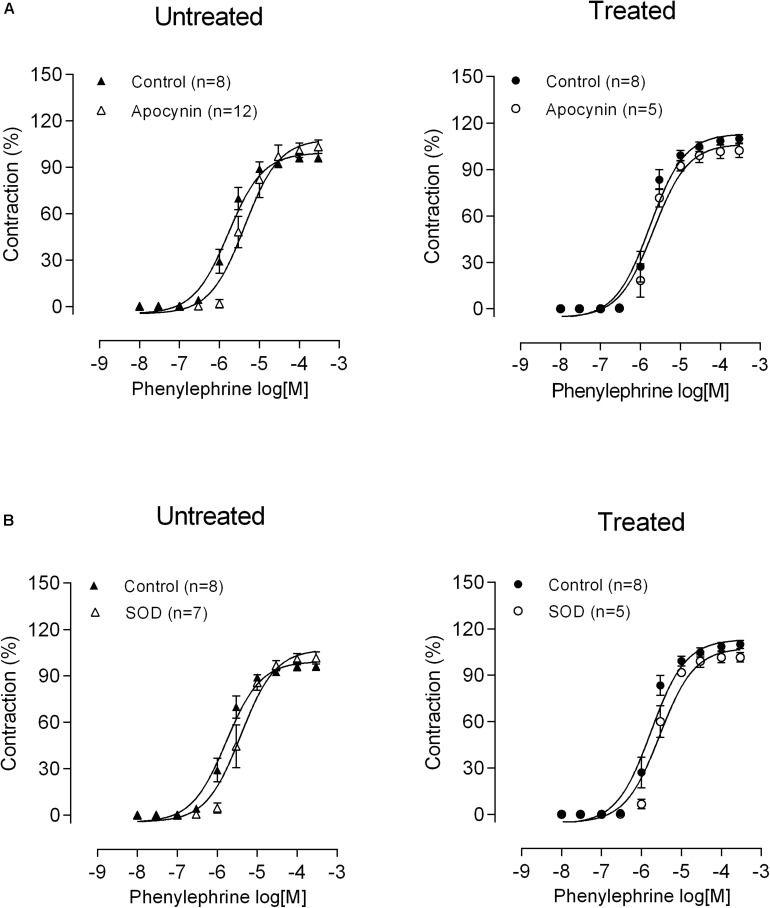
Role of oxidative stress in altered phenylephrine responses after lead treatment. The effects of **(A)** apocynin and **(B)** SOD on the concentration-response curve to phenylephrine in mesenteric rings from untreated and treated rats; Data are expressed as the mean ± SEM. *P* > 0.05 versus control. n denotes the number of animals used.

Another possibility to increase vasoconstriction of MRA could be the production of H_2_O_2_ *via* NADPH oxidase and SOD. To determine whether the changes in vascular reactivity observed in the mesenteric rings after lead exposure were linked to an increase in O_2_^–^ production, the effects of the NADPH oxidase inhibitor apocynin and the superoxide anion scavenger SOD were assessed. Neither apocynin (30 μM) nor SOD (150 U mL^–1^) modified the vasoconstrictor responses to phenylephrine in either experimental group (Figures 3A,B and Table 1). Altogether, these findings suggest that chronic treatment with low concentrations of lead do not induce oxidative stress *via* NADPH oxidase, which could contribute to the increased reactivity of MRA to phenylephrine.

### Role of Lead Effects on the Cyclooxygenase Pathway

**FIGURE 4:**
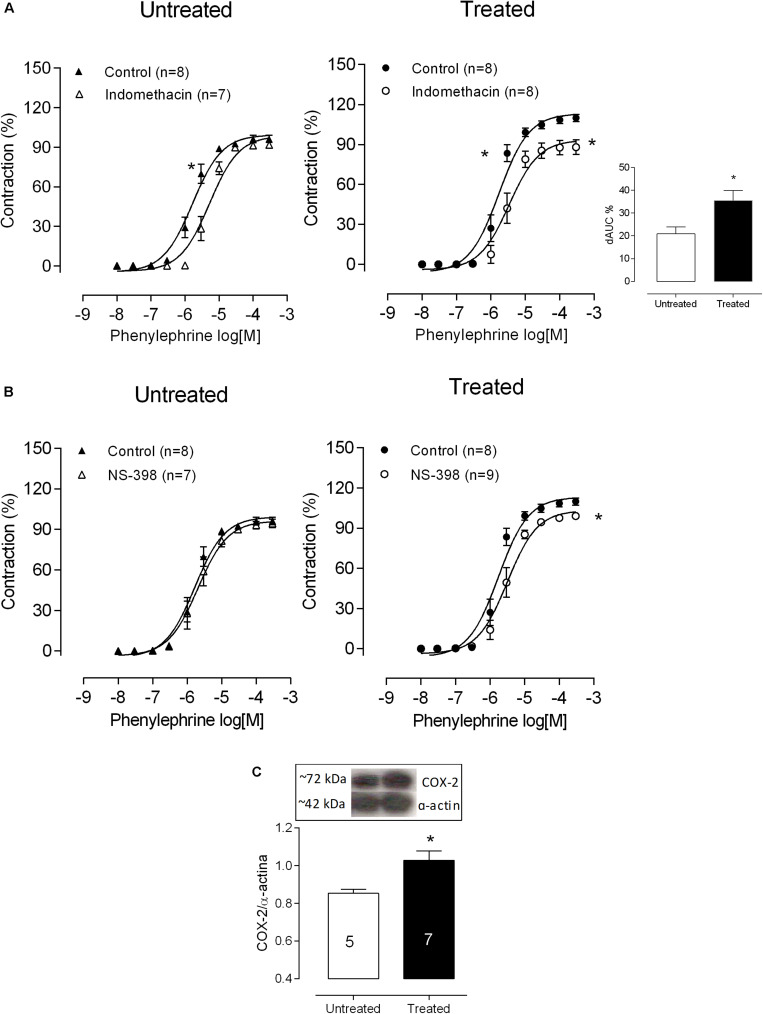
Role of COX-2-derived prostanoids in altered phenylephrine responses after lead treatment. The effects of **(A)** the non-selective COX inhibitor indomethacin and **(B)** the selective COX-2 inhibitor NS398 on the concentration-response curve to phenylephrine in mesenteric rings from untreated and treated rats. The insert shows differences in the area under the concentration-response curves (dAUC) in the presence and absence of indomethacin. *P < 0.05 versus control using two-way ANOVA and Bonferroni *post hoc* test or Student’s *t*-test. **(C)** Densitometric analysis of Western Blots for COX-2 protein expression in mesenteric arteries from untreated and treated rats. Representative blots are also shown. *P < 0.05 versus untreated by Student’s *t*-test. Data are expressed as the mean ± SEM. n denotes the number of animals used.

To investigate the putative role of prostanoids, mesenteric rings were incubated with indomethacin (10 μM), a non-specific COX inhibitor. The response to phenylephrine was reduced in both experimental groups. However, in preparations from lead-treated rats this effect was enhanced when compared to controls, as demonstrated by the dAUC (Figure 4A and Table 1). These results suggest that the enhanced vasoconstrictor responses depend on involvement of vasoconstrictor prostanoids in.

**FIGURE 5:**
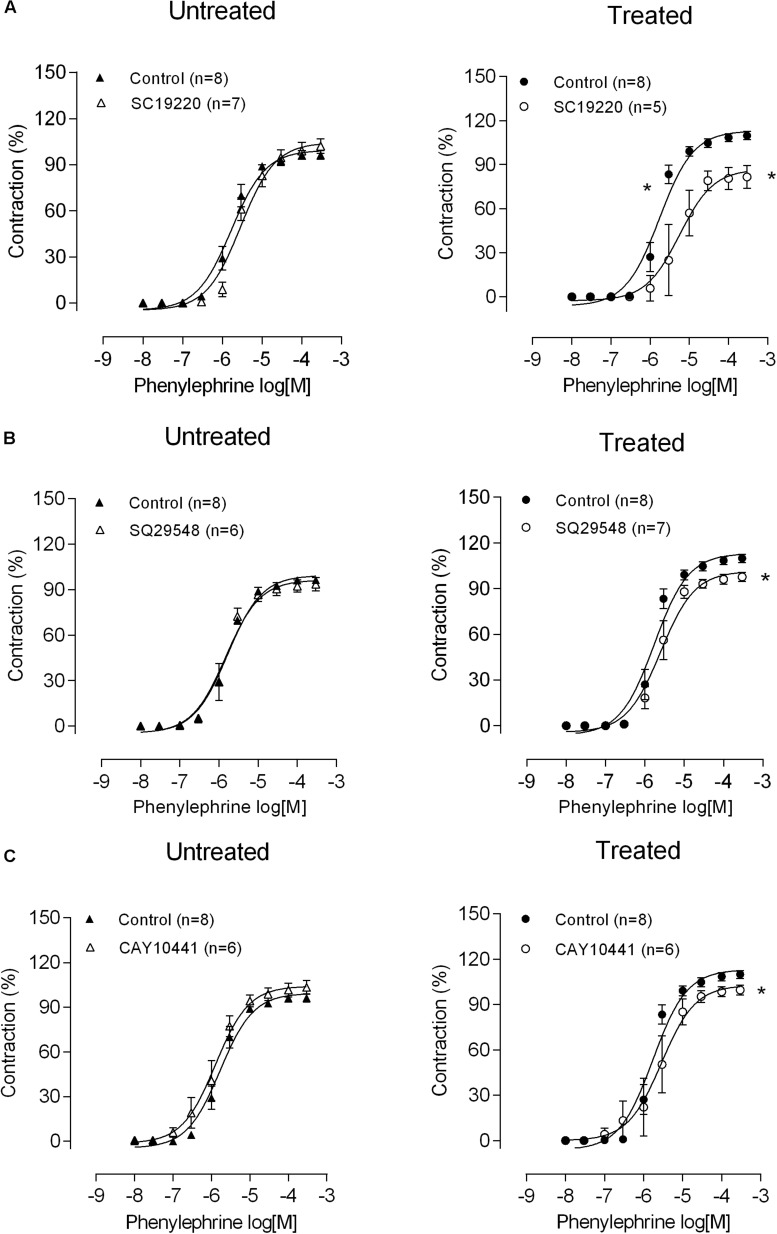
Role of prostanoidsin altered phenylephrine responses after lead treatment. The effect of **(A)** the EP1 antagonist SC19220, **(B)** the TXA2 receptor antagonist SQ29548, and **(C)** the IP receptor antagonist CAY10441 on the concentration-response curve to phenylephrine in mesenteric rings from untreated and treated rats. *P < 0.05 versus control using two-way ANOVA and Bonferroni *post hoc* test. Data are expressed as the mean ± SEM. n denotes the number of animals used and is indicated in parentheses.

We also investigated whether lead effects were resulting from the involvement of COX-2, prostaglandin E_2_ (PGE2), thromboxane A_2_ (TXA2) and prostacyclin I_2_ (PGI2), products of COX-2. Then, mesenteric rings were incubated with the COX-2 inhibitor NS398 (1 μM), an antagonist of the EP1 receptor (SC19220, 1 μM), the TP receptor antagonist SQ 29548 (1 μM) and the IP receptor antagonist CAY10441 (100 nM). NS398 had no effects on phenylephrine responses of control mesenteric segments. However, in arteries from lead-treated rats, NS398 reduced phenylephrine contraction (Figure 4B and Table 1), suggesting that COX-2 was playing a role in the vascular effects of lead. In agreement, COX-2 protein expression increased in vessels from lead-treated rats (Figure 4C and Table 1). SC19220, SQ29548, and CAY10441 did not change the vascular reactivity to phenylephrine in the control mesenteric rings, but in the lead exposure group the phenylephrine-induced response was reduced, as shown in Figures 5A–C and Table 1). Jointly, these findings suggest that chronic treatment with low concentrations of lead enhances the production of COX-2 derived vasoconstrictor prostanoids. Therefore, thromboxane A_2_, prostaglandin E_2_ and prostacyclin I_2_ might contribute to impair the vascular function of the MRA from lead-treated rats.

### Role of the Renin-Angiotensin System in the Effect of Lead on Vasoconstrictor Responses

**FIGURE 6:**
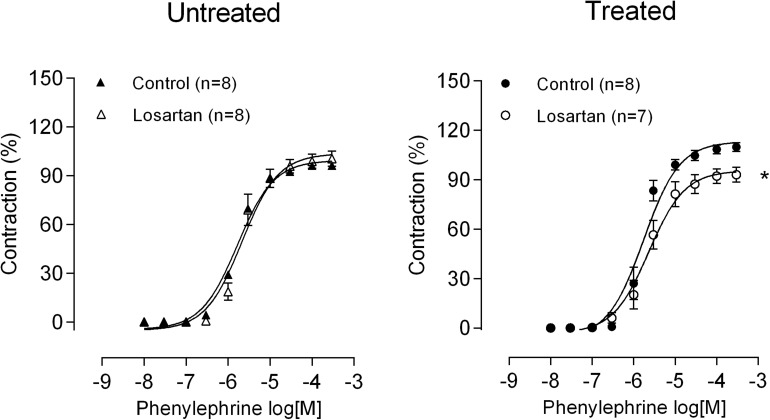
Role of angiotensin II in the altered phenylephrine responses after lead treatment. The effect of the selective AT1antagonistlosartan on the concentration-response curve to phenylephrine in mesenteric rings from untreated and treated rats. *P < 0.05 versus control using two-way ANOVA and Bonferroni *post hoc* test. Data are expressed as the mean ± SEM. n denotes the number of animals used and is indicated in parentheses.

Another mechanism that could play a role regarding lead effects could be the stimulation of AT1 receptors. This receptor has a vasoconstrictor action, but it also stimulates COX expression (Briones and Touyz, 2010). To investigate the putative involvement of the renin-angiotensin system in the lead effects on the alterations of vascular reactivity to phenylephrine, losartan (10 μM) an AT1 receptors blocker was used. As shown in Figure 6, losartan reduced the vasoconstrictor response induced by phenylephrine in MRA from lead-treated rats but not in those from control rats (Figure 6 and Table 1). This result suggests that lead exposure might enhance the activity of the local renin-angiotensin system and reinforces the hypothesis that the AT1 receptors might be involved in the rise of MRA vasoconstrictor responses and arterial blood pressure in lead-treated rats.

## Discussion

The main results reported here are that exposure to low doses of lead increases the peripheral resistance as shown by the increased vascular tone of MRA. Such tone increment depends on the enhanced production of COX-2-derived prostanoids; in addition, a possible role for angiotensin II is also suggested. The treatment regime used in this study attained blood lead concentrations of 21.7 μg/dL, a value that is lower than the reference values (Agency for Toxic Substances and Disease Registry [ATSDR], 2019), and this concentration has been shown to be sufficient to increase systolic blood pressure (Simões et al., 2015).

The consequence of lead exposure that we observed in resistance arteries is an increased vasoconstrictor response to phenylephrine, which might explain, at least in part, the hypertensinogenic effect of lead. Different from conductance arteries the results of endothelial removal and pharmacological interventions showed that NO bioavailability is preserved in the MRAs and that there is no involvement of oxidative stress in the observed increased reactivity of MRA. In addition, results from these interventions suggest that COX-2 and the renin–angiotensin system are involved in the effects of lead in resistance arteries, and consequently, in the increased vascular peripheral tone.

Hypertension is a chronic disease of multifactorial etiology, and it is considered an important public health problem because it is one of the cardiovascular risk factors (Yazbeck et al., 2009). Previous reports suggested that high blood lead levels correlates with hypertension development in animals and humans (Victery et al., 1982; Carmignani et al., 1999; Andrzejak et al., 2004; Patrick, 2006; Kosnett et al., 2007; Navas-Acien et al., 2007; Silveira et al., 2014; Simões et al., 2015). It is important to emphasize that the value found in exposed persons, accepted by agencies, as the Agency for Toxic Substances and Disease Registry (ATSDR) considers the reference blood lead concentration to be 60 μg/dL (Patrick, 2006; Kosnett et al., 2007). However, in this model of exposure, the blood lead concentration was 21.7 ± 2.38 μg/dL (Simões et al., 2015), which is less than 60 μg/dL. Moreover, despite the existence of reports that show the toxic effects of this metal, the effects of exposure to low doses of lead on the vascular function has been described in conductance arteries but effects on resistance arteries are not yet clear.

In this study, we used the mesenteric resistance arteries, which play a key role in the total vascular resistance and therefore in the maintenance of an increased blood pressure (Mulvany and Aalkjaer, 1990). We observed that lead exposure increased the maximum contractile response to phenylephrine without changing the sensitivity. In agreement with our findings, Skoczynska et al. (1986) showed enhanced pressor responses to norepinephrine in isolated mesenteric arteries in rats exposed to lead for 5 weeks (50.0 mg/kg/day gavage).

To ascertain the mechanisms by which lead promotes alterations in the reactivity of the mesenteric resistance arteries, we investigated the role of the negative modulation of the endothelium. In mesenteric rings with denuded endothelium, the reactivity to phenylephrine was similarly increased in both experimental groups. Then, we used L-NAME to clarify the likely role of NO in the effect of lead on the contractile responses to phenylephrine. The findings indicated that lead incubation did not alter the endothelial modulation induced by NO on the vasoconstrictor responses in resistance arteries. In contrast, our laboratory demonstrated that low doses of lead reduce the bioavailability of NO in the aorta as a consequence of increased ROS production (Silveira et al., 2014; Simões et al., 2015). Accordingly, with our present findings, the endothelium-dependent and endothelium-independent relaxation induced by ACh and the known NO donor sodium nitroprusside, respectively, did not change after 30 days of lead exposure. Our results suggest that lead induces different effects depending on the vascular bed.

Oxidative stress has been reported to contribute to the altered responses in different vessels after exposure to several heavy metals (Wiggers et al., 2008; Angeli et al., 2013). However, our findings suggested that treatment with apocynin or SOD did not reverse lead effects on the vascular reactivity to phenylephrine in the MRA, suggesting that subjected to lead exposure, ROS release did not contribute to vascular dysfunction. By contrast, we recently showed that treatment with lead increased superoxide anion production in both aorta and in VSMCs (Simões et al., 2015). This difference might be explained by the fact that conduction arteries are more NO-dependent, while resistance arteries are not (Brandes et al., 2000; McNeish et al., 2002; Freitas et al., 2003).

Prostanoids derived from COX-2 have also been involved in the vascular lead effects (Silveira et al., 2014) as well as the effects of other heavy metals like mercury (Peçanha et al., 2010). The presence of COX-2 in the media layer of the arteries mainly contributes to the changing vascular tone (Bishop-Bailey et al., 1999), and increased vascular expression of COX-2 is often in association with hypertension (Hernanz et al., 2014). The reduction of the increased vasoconstrictor response to phenylephrine by COX blockade with indomethacin, which is observed only in lead-treated rats, suggests that vasoconstrictor prostanoids play a role in the effects of lead. The selective COX-2 inhibitor NS398 decreased the vasoconstrictor responses induced by phenylephrine in the lead-treated animals but not in controls, proponing that prostanoids that contribute to the lead effect are produced by the inducible isoform of COX-2.

The greater participation of COX-2-derived products observed after lead treatment could be associated with the upregulation of this COX isoform. In this sense, we found an increase of COX-2 protein expression in mesenteric arteries from lead-treated rats compared to untreated rats, reinforcing the functional data. Accordingly, we recently reported that the exposure of vascular smooth muscle cells to lead (20 μg/dl) increased COX-2 at both the mRNA and protein levels (Simões et al., 2015). In the tail vascular bed, it has been shown that acute lead exposure has effects on the endothelium, releasing COX-derived vasoconstrictors (Silveira et al., 2010), and that COX-2 activation contributes to vascular changes after chronic lead exposure in aorta segments (Silveira et al., 2014; Simões et al., 2015). As to the best of our knowledge, this is the first report to show the contribution of COX-2 to the altered responses in resistance vessels produced by low level lead exposure.

Then, we aimed to elucidate the nature of the COX-derived vasoconstrictors involved in the altered phenylephrine responses. The first step was to investigate COX-derived prostanoids. Incubations with EP1 receptor antagonist SC19220, the TP receptor antagonist SQ 29548, and the IP receptor antagonist CAY 10441 decreased phenylephrine contractile responses only in lead-treated animals, thus suggesting the participation of COX-derived prostanoids in the effects of lead. It’s well known, that PGI2 promotes vasodilation in various vascular beds by stimulating prostacyclin receptors (IP) and thereby increasing the intracellular cyclic-AMP concentration (Wise and Jones, 1996). However, PGI2 can trigger a biphasic vasomotor response, in which lower concentrations cause relaxation, while higher concentrations cause contraction through the activation of TP receptors (Levy, 1980; Williams et al., 1994; Zhao et al., 1996; Blanco-Rivero et al., 2005; Xavier et al., 2008, 2009). Taken together, our findings suggest for the first time the participation TxA2, PGE2 and PGI2 on changes of endothelial function produced by lead in resistance vessels and this endothelial dysfunction can be associated with cardiovascular risk factors.

It is known that angiotensin II contributes to the development of hypertension by its vasoconstrictor action and modulating prostaglandin production as a consequence of the regulation of COX-2 expression (Ohnaka et al., 2000; Harris et al., 2004; Alvarez et al., 2007; Beltrán et al., 2009; Hernanz et al., 2014). In cultured rat vascular smooth muscle cells (Ohnaka et al., 2000; Hu et al., 2002) and in adventitial fibroblasts (Beltrán et al., 2009), angiotensin II induces COX-2 expression. Furthermore, previous reports have revealed that lead exposure increases the activity of the local and systemic renin–angiotensin system (Fiorim et al., 2011; Simões et al., 2011; Silveira et al., 2014). We found that the increased vascular reactivity in the mesenteric rings after treatment with lead could be a consequence of the activation of SRA, as suggested by the losartan blockade of the vasoconstrictor effects observed only in segments from treated animals. Therefore, we suggest that the increased local angiotensin II production might be responsible, at least in part, for the increased COX-2 activity after chronic lead exposure.

## Conclusion

In conclusion, we demonstrated for the first time that the chronic exposure to small doses of lead increases the reactivity of the peripheral vasculature. Also, such effects are different from lead actions on conductance arteries that involves reduction of NO bioavailability and oxidative stress. In addition, lead treatment enhances the liberation of COX-2-derived vasoconstrictor prostanoids. In association with this increased COX-2 activity it occurs an increased activation of the renin-angiotensin system caused by lead treatment. These actions might help to explain the increased vasoconstrictor responses induced by lead exposure. In addition, it must be emphasized that the present findings reinforce the significance of lead as a hazardous environmental contaminant. It harms the organism producing undesirable effects to the cardiovascular system, which might contribute to the genesis and maintenance of hypertension. These findings strongly support the viewpoint that the concentration of lead, considered to be safe, must be reduced.

## Data Availability Statement

The original contributions presented in the study are included in the article/Supplementary Material, further inquiries can be directed to the corresponding author/s.

## Ethics Statement

The animal study was reviewed and approved by Ethics Committee in Animal Research of the Federal University of Espírito Santo (CEUA 063/2011).

## Author Contributions

MRS, BA, MA, MS, and DV participated in the study design. MRS wrote first draft of the manuscript. MRS and BA performed the experiments. MRS and DV conducted the data interpretation and analyses. MRS, BA, MA, MS, and DV reviewed the manuscript submitted for publication. All authors revised and approved the final version of the manuscript.

## Conflict of Interest

The authors declare that the research was conducted in the absence of any commercial or financial relationships that could be construed as a potential conflict of interest.

## References

[B1] Agency for Toxic Substances and Disease Registry [ATSDR] (2019). *Toxicological profile for lead. (Draft for Public Comment).* (Atlanta, GA: U.S. Department of Health and Human Services), 1–561.

[B2] Alvarez Y. Briones A. M. Balfagón G. Alonso M. J. Salaceis M. (2005). Hypertension increases the participation of vasoconstrictor prostanoids from cyclooxigenase-2 in phenylephine responses. *J. Hypertens.* 23 767–777. 10.1097/01.hjh.0000163145.12707.63 15775781

[B3] Alvarez Y. Pérez-Girón J. V. Hernánz R. Briones A. M. García-Redondo A. Beltran A. (2007). Losartan reduces the increased participation of cyclooxygenase-2-derived products in vascular responses of hypertensive rats. *J. Pharmacol. Exp. Ther.* 321 381–388. 10.1124/jpet.106.115287 17244722

[B4] Andrzejak R. Poreba R. Derkacz A. (2004). Effect of chronic lead poisoning on the parameters of heart rate variability. *Med. Med. Pr.* 55 139–144. 15524081

[B5] Angeli J. K. Pereira C. A. C. de Oliveira F. T. Stefanon I. Padilha A. S. Vassallo D. V. (2013). Cadmium exposure induces vascular injury due to endothelial oxidative stress: the role of local angiotensin II and COX-2. *Free Radic. Biol. Med.* 65 838–848. 10.1016/j.freeradbiomed.2013.08.167 23973752

[B6] Beltrán A. E. Briones A. M. Garcia-Redondo A. B. Rodríguez C. Miguel M. Alvarez Y. (2009). p38 MAPK contributes to angiotensin II-induced COX-2 expression in aortic fibroblasts from normotensive and hypertensive rats. *J. Hypertens.* 27 142–154. 10.1097/hjh.0b013e328317a730 19145780

[B7] Bishop-Bailey D. Hla T. Mitchell J. A. (1999). Cyclo-oxygenase-2 in vascular smooth muscle. *Int. J. Mol. Med.* 3 41–48. 10.3892/ijmm.3.1.41 9864384

[B8] Blanco-Rivero J. Cachofeiro V. Lahera V. Aras-Lopez R. Márquez-Rodas I. Salaices M. (2005). Participation of prostacyclin in endothelial dysfunction induced by aldosterone in normotensive and hypertensive rats. *Hypertension* 46 107–112. 10.1161/01.HYP.0000171479.36880.17 15956108

[B9] Brandes R. P. Schmitz-Winnenthal F. H. Feletou M. Godecke A. Hunag P. L. Vanhoutte P. M. (2000). An endothelium-derivedhyperpolarizing factor distinct from NO and prostacyclin is a major endotheliumdependentvasodilator in resistance vessels of wild-type and endothelial NOsynthase knockout mice. *Proc. Natl. Acad. Sci. U.S.A.* 97 9747–9752. 10.1073/pnas.97.17.9747 10944233 PMC16936

[B10] Briones A. M. Touyz R. M. (2010). Oxidative stress and hypertension: current concepts. *Curr. Hypertens. Rep.* 12 135–142. 10.1007/s11906-010-0100-z 20424957

[B11] Carmignani M. Boscolo P. Poma A. Volpe A. R. (1999). Kininergic system and arterial hypertension following chronic exposure to inorganic lead. *Immunopharmacology* 44 105–110. 10.1016/s0162-3109(99)00115-0 10604532

[B12] Farmand F. Ehdaie A. Roberts C. K. Sindhu R. K. (2005). Lead-induced dysregulation of superoxide dismutases, catalase, glutathione peroxidase, and guanylate cyclase. *Environ. Res.* 98 33–39. 10.1016/j.envres.2004.05.016 15721881

[B13] Fiorim J. Ribeiro R. F. Jr. Azevedo B. F. Simões M. R. Padilha A. S. Stefanon I. (2012). Activation of K+ channels and Na+/K+ ATPase prevents aortic endothelial dysfunction in 7-day lead-treated rats. *Toxicol. Appl. Pharmacol.* 262 22–31. 10.1016/j.taap.2012.04.015 22546088

[B14] Fiorim J. Ribeiro Junior R. F. Silveira E. A. Padilha A. S. Vescovi M. V. de Jesus H. C. (2011). Low-level lead exposure increases systolic arterial pressure and endothelium-derived vasodilator factors in rat aortas. *PLoS One* 6:e17117. 10.1371/journal.pone.0017117 21364929 PMC3045404

[B15] Freis E. D. (1973). Age, race and sex and other indices of risk in hypertension. *Am. J. Med.* 55 275–280. 10.1016/0002-9343(73)90129-0 4746555

[B16] Freitas M. R. Schott C. Corriu C. Sassard J. Stoclet J. C. Andriantsitohaina R. (2003). Heterogeneity of endothelium-dependent vasorelaxation in conductance and resistance arteries from Lyon normotensive and hypertensive rats. *J. Hypertens.* 21 1505–1512. 10.1097/00004872-200308000-00014 12872044

[B17] Gonick H. C. Ding Y. Bondy S. C. Ni Z. Vaziri N. D. (1997). Lead-induced hypertension: interplay of nitric oxide and reactive oxygen species. *Hypertension* 30 1487–1492. 10.1161/01.hyp.30.6.1487 9403571

[B18] Grizzo L. T. Cordelline S. (2008). Perinatal lead exposure affects nitric oxide and cyclooxygenase pathways in aorta of weaned rats. *Toxicol. Sci.* 103 207–214. 10.1093/toxsci/kfn018 18234738

[B19] Harrap S. B. (1994). Hypertension: genes versus environment. *Lancet* 344 171 10.1016/s0140-6736(94)92762-6 7912770

[B20] Harris R. C. Zhang M. Z. Cheng H. F. (2004). Cyclooxygenase-2 and the renal renin-angiotensin system. *Acta Physiol. Scand.* 181 543–547. 10.1111/j.1365-201X.2004.01329.x 15283769

[B21] Hernanz R. Briones A. M. Salaices M. Alonso M. J. (2014). New roles for old pathways? A circuitous relationship between reactive oxygen species and cyclo-oxygenase in hypertension. *Clin. Sci.* 126 111–121. 10.1042/CS20120651 24059588

[B22] Hu Z. W. Kerb R. Shi X. Y. Wei-Lavery T. Hoffman B. B. (2002). Angiotensin II increases expression of cyclooxygenase-2: implications for the function of vascular smooth muscle cells. *J. Pharmacol. Exp. Ther.* 303 563–573. 10.1124/jpet.102.037705 12388637

[B23] Khalil-Manesh F. Gonick H. C. Weiler E. W. Prins B. Weber M. A. Purdy R. E. (1993). Lead-induced hypertension: possible role of endothelial factors. *Am. J. Hypertens.* 6 723–729. 10.1093/ajh/6.9.723 8110424

[B24] Kosnett M. J. Wedeen R. P. Rothenberg S. J. Hipkins K. L. Materna B. L. Schwartz B. S. (2007). Recommendations for medical management of adult lead exposure. *Environ. Health Perspect.* 115 463–471. 10.1289/ehp.9784 17431500 PMC1849937

[B25] Levy J. V. (1980). Prostacyclin-induced contraction of isolated aortic strips from normal and spontaneously hypertensive rats (SHR). *Prostaglandins* 19 517–525. 10.1016/s0090-6980(80)80002-5 6992230

[B26] Martínez-Revelles S. Avendano M. S. García-Redondo A. B. Álvarez Y. Aguado A. Pérez-Girón J. V. (2013). Reciprocal relationship between reactive oxygen species and cyclooxygenase-2 and vascular dysfunction in hypertension. *Antioxid. Redox Signal.* 18 51–65. 10.1089/ars.2011.4335 22671943

[B27] McNeish A. J. Wilson W. S. Martin W. (2002). Ascorbate blocks endotheliumderivedhyperpolarizing factor (EDHF)-mediated vasodilation in the bovine ciliaryvascular bed and rat mesentery. *Br. J. Pharmacol.* 135 1801–1809. 10.1038/sj.bjp.0704623 11934822 PMC1573289

[B28] Mulvany M. J. Aalkjaer C. (1990). Structure and function of small arteries. *Physiol. Rev.* 70 921–961. 10.1152/physrev.1990.70.4.921 2217559

[B29] Mulvany M. J. Halpern W. (1977). Contractile properties of small arterial resistance vessels in spontaneously hypertensive and normotensive rats. *Circ. Res.* 41 19–26. 10.1161/01.res.41.1.19 862138

[B30] Navas-Acien A. Guallar E. Silbergeld E. K. Rothenberg S. J. (2007). Lead exposure in cardiovascular disease –A systematic review. *Environ. Health Perspect.* 115 472–482. 10.1289/ehp.9785 17431501 PMC1849948

[B31] Ohnaka K. Numaguchi K. Yamakawa T. Inagami T. (2000). Induction of cyclooxygenase-2 by angiotensin II in cultured rat vascular smooth muscle cells. *Hypertension* 35 68–75. 10.1161/01.hyp.35.1.68 10642277

[B32] Patrick L. (2006). Lead toxicity part II: the role of free radical damage and the use of antioxidants in the pathology and treatment of lead toxicity. *Altern. Med. Rev.* 11 114–127. 16813461

[B33] Peçanha F. M. Wiggers G. A. Briones A. M. Pérez-Girón J. V. Miguel M. García-Redondo A. B. (2010). The role of cyclooxygenase (COX)-2 derived prostanoids on vasoconstrictor responses to phenylephrine is increased by exposure to low mercury concentration. *J. Physiol. Pharmacol.* 61 29–36. 20228412

[B34] Prozialeck W. C. Edwards J. R. Nebert D. W. Woods J. M. Barchowsky A. Atchison W. D. (2008). The vascular system as a target of metal toxicity. *Toxicol. Sci.* 102 207–218. 10.1093/toxsci/kfm263 17947343 PMC2752624

[B35] Rahman S. Khalid N. Zaidi Z. H. Ahmad S. Iqbal M. Z. (2006). Non-occupational lead exposure and hypertension in Pakistani adults. *J. Zhejiang Univ. Sci. B* 7 732–737. 10.1631/jzus.2006.b0732 16909475 PMC1559805

[B36] Shakir S. K. Azizullah A. Murad W. Daud M. K. Nabeela F. Rahman H. (2017). Toxic metal pollution in pakistan and its possible risks to public health. *Rev. Environ. Contam. Toxicol.* 242 1–60. 10.1007/398_2016_9 27464847

[B37] Silveira E. A. Lizardo J. H. F. Souza L. P. Stefanon I. Vassallo D. V. (2010). Acute lead-induced vasoconstriction in vascular beds of isolated perfused rat tails in endothelium dependent. *Braz. J. Med. Biol. Res.* 43 492–499. 10.1590/s0100-879x2010007500027 20396857

[B38] Silveira E. A. Siman F. D. de Oliveira F. T. Vescovi M. V. Furieri L. B. Lizardo J. H. (2014). Low-dose chronic lead exposure increases systolic arterial pressure and vascular reactivity of rat aortas. *Free Radic. Biol. Med.* 67 366–376. 10.1016/j.freeradbiomed.2013.11.021 24308934

[B39] Simões M. R. Aguado A. Fiorim J. Silveira E. A. Azevedo B. F. Toscano C. M. (2015). MAPK pathway activation by chronic lead-exposure increases vascular reactivity through oxidative stress/cyclooxygenase-2-dependent pathways. *Toxicol. Appl. Pharmacol.* 283 127–138. 10.1016/j.taap.2015.01.005 25596430

[B40] Simões M. R. Preti S. C. Azevedo B. F. Fiorim J. Freire D. D. Jr. Covre E. P. (2016). Low-level chronic lead exposure impairs neural control of blood pressure and heart rate in rats. *Cardiovasc. Toxicol.* 17 190–199. 10.1007/s12012-016-9374-y 27272938

[B41] Simões M. R. Ribeiro Júnior R. F. Vescovi M. V. de Jesus H. C. Padilha A. S. Stefanon I. (2011). Acute lead exposure increases arterial pressure: role of the renin-angiotensin system. *PLoS One* 6:e18730. 10.1371/journal.pone.0018730 21494558 PMC3073979

[B42] Skoczynska A. Juzwa W. Smolik R. Szechinski J. Behal F. J. (1986). Response of the cardiovascular system to catecholamines in rats given small doses of lead. *Toxicology* 39 275–289. 10.1016/0300-483x(86)90028-4 3705089

[B43] Trott D. W. Harrison D. G. (2014). The immune system in hypertension. *Adv. Physiol. Educ.* 38 20–24. 10.1152/advan.00063.2013 24585465 PMC4459918

[B44] Vassallo D. V. Simões M. R. Furieri L. B. Fioresi M. Fiorim J. Almeida E. A. (2011). Toxic effects of mercury, lead and gadolinium on vascular reactivity. *Braz. J. Med. Biol. Res.* 44 939–946. 10.1590/s0100-879x2011007500098 21845340

[B45] Vaziri N. D. (2002). Pathogenesis of lead-induced hypertension: role of oxidative stress. *J. Hypertens. Suppl.* 20 S15–S20. 12184052

[B46] Vaziri N. D. Ding Y. Ni Z. (2001). Compensatory up-regulation of nitric-oxide synthase isoforms in lead-induced hypertension; reversal by a superoxide dismutase-mimetic drug. *J. Pharmacol. Exp. Ther.* 298 679–685. 11454931

[B47] Vaziri N. D. Gonick H. C. (2008). Cardiovascular effects of lead exposure. *Indian J. Med. Res.* 128 426–435. 19106438

[B48] Victery W. Vander A. J. Shulak J. M. Schoeps P. Julius S. (1982). Lead, hypertension, and the renin-angiotensin system in rats. *J. Lab. Clin. Med.* 99 354–362. 7057062

[B49] Virdis A. Bacca A. Colucci R. Duranti E. Fornai M. Materazzi G. (2013). Endothelial dysfunction in small arteries of essential hypertensive patients: role of cyclooxygenase-2 in oxidative stress generation. *Hypertension* 62 337–344. 10.1161/HYPERTENSIONAHA.111.00995 23734008

[B50] Vupputuri S. He J. Muntner P. Bazzano L. A. Whelton P. K. Batuman V. (2003). Blood lead level is associated with elevated blood pressure in blacks. *Hypertension* 41 463–468. 10.1161/01.hyp.0000055015.39788.29 12623944

[B51] Weiler E. Khalil-Manesh F. Gonick H. C. (1990). Effects of lead and a low-molecular-weight endogenous plasma inhibitor on the kinetics of sodium-potassium-activated adenosine triphosphatase and potassium-activated p-nitrophenylphosphatase. *Clin. Sci.* 79 185–192. 10.1042/cs0790185 2167808

[B52] Wiggers G. A. Peçanha F. M. Briones A. M. Pérez-Girón J. V. Miguel M. Vassallo D. V. (2008). Low mercury concentrations cause oxidative stress and endothelial dysfunction in conductance and resistance arteries. *Am. J. Physiol. Heart Circ. Physiol.* 295 H1033–H1043. 10.1152/ajpheart.00430.2008 18599595

[B53] Williams S. P. Dorn G. W. Rapoport R. M. (1994). Prostaglandin I2 mediates contraction and relaxation of vascular smooth muscle. *Am. J. Physiol.* 267(2 Pt 2), H796–H803. 10.1152/ajpheart.1994.267.2.H796 8067435

[B54] Wise H. Jones R. L. (1996). Focus on prostacyclin and its novel mimetics. *Trends Pharmacol. Sci.* 17 17–21. 10.1016/0165-6147(96)81565-3 8789354

[B55] Wong S. L. Wong W. T. Tian X. Y. Lau C. W. Huang Y. (2010). Prostaglandins in action indispensable roles of cyclooxygenase-1 and -2 in endothelium-dependent contractions. *Adv. Pharmacol.* 60 61–83. 10.1016/B978-0-12-385061-4.00003-9 21081215

[B56] Xavier F. E. Aras-López R. Arroyo-Villa I. del Campo L. Salaices M. Rossoni L. V. (2008). Aldosterone induces endothelial dysfunction in resistance arteries from normotensive and hypertensive rats by increasing thromboxane A2 and prostacyclin. *Br. J. Pharmacol.* 154 1225–1235. 10.1038/bjp.2008.200 18500359 PMC2483383

[B57] Xavier F. E. Blanco-Rivero J. Ferrer M. Balfagón G. (2009). Endothelium modulates vasoconstrictor response to prostaglandin I2 in rat mesenteric resistance arteries: interaction between EP1 and TP receptors. *Br. J. Pharmacol.* 158 1787–1795. 10.1111/j.1476-5381.2009.00459.x 19891662 PMC2801220

[B58] Xie Y. Chiba M. Shinohara A. Watanabe H. Inaba Y. (1998). Studies on lead–binding protein and interaction between lead and selenium in the human erythrocytes. *Ind. Health* 36 234–239. 10.2486/indhealth.36.234 9701901

[B59] Yazbeck C. Thiebaugeorges O. Moureau T. Goua V. Debotte G. Sahuquillo J. (2009). Maternal blood lead levels and the risck of pregnancy-induced hypertension: the EDEN cohort study. *Environ. Health Perspect.* 117 1527–1530. 10.1289/ehp.0800488 20019901 PMC2790505

[B60] Zhao Y. J. Wang J. Tod M. L. Rubin L. J. Yuan X. J. (1996). Pulmonary vasoconstrictor effects of prostacyclin in rats: potential role of thromboxane receptors. *J. Appl. Physiol.* 81 2595–2603. 10.1152/jappl.1996.81.6.2595 9018511

