# Supplementary figures and images for: Chronic Low-Level Lead Exposure Increases Mesenteric Vascular Reactivity: Role of Cyclooxygenase-2-Derived Prostanoids
Source: Front Physiol. 2021 Jan 7;11:590308. doi: 10.3389/fphys.2020.590308 (PMC7818781; doi:10.3389/fphys.2020.590308)

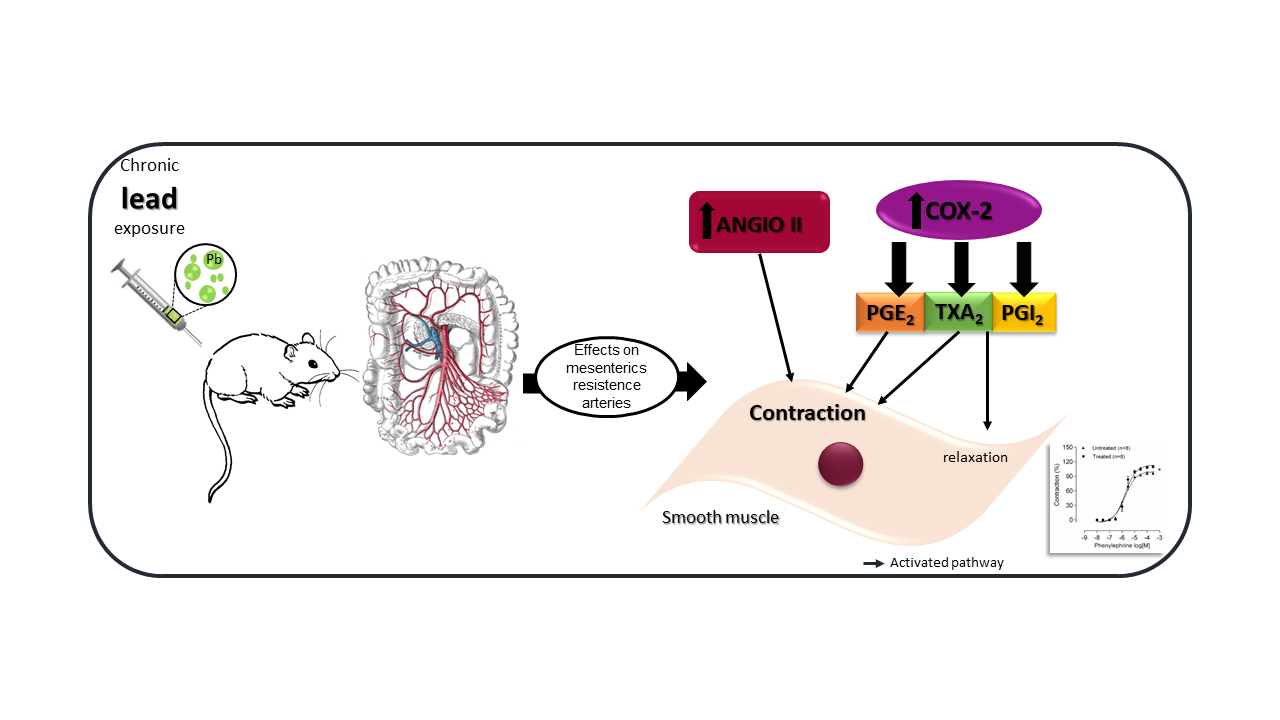

Supplement: Supplementary file 1 [file Image_1.TIF]
